# Quantitative bioluminescence imaging of transgene expression in intact porcine antral follicles *in vitro*

**DOI:** 10.1186/1477-7827-12-11

**Published:** 2014-01-30

**Authors:** Song-yi Jung, Scott T Willard

**Affiliations:** 1 Department of Biochemistry, Molecular Biology, Entomology, and Plant Pathology, Mississippi State University, 32 Creelman Street-Room 402 Dorman Hall, Mississippi, MS, USA

**Keywords:** Bioluminescence imaging, *in vitro* intact antral follicle culture, Lipid mediated gene transfer method

## Abstract

**Background:**

The porcine oocyte maturation *in vivo* occurs within the ovarian follicle and is regulated by the interactions between oocytes and surrounding follicular components, including theca, granulosa, and cumulus cells, and follicular fluid. Therefore, the antral follicle is an essential microenvironment for efficient oocyte maturation and its developmental competence. Quantitative bioluminescence imaging of firefly luciferase reporter genes in an intact antral follicle would allow investigation of changes in cellular and molecular events and in the context of the whole follicles. In this study, we investigate factors influencing bioluminescence measurements as a first step towards developing a new bioluminescence imaging system for intact antral follicles.

**Methods:**

We analyzed the time course of bioluminescence emitted from transfected living intact follicles using a cationic lipid mediated gene transfer method with increasing doses (1-3 μg) of firefly luciferase reporter gene (pGL4). In addition, a standard luciferase assay was used to confirm the luciferase expression in granulosa cells in the transfected intact antral follicles. Finally, the dose effects of substrate, D-luciferin, were determined for optimal quantitative bioluminescence imaging of intact porcine antral follicles *in vitro*.

**Results:**

The level of luciferase activity of follicles with 3 μg pGL4 was significantly (*P* < 0.05) greater than the 1 μg and 2 μg groups at 1 min after D-luciferin injection. The bioluminescence intensity of transfected follicles reached a peak at 1 min, and then it was significantly (*P* < 0.05) reduced within 2 min after injection of D-luciferin; with the level of bioluminescence emission remained constant from 2.5 to 10 min. The bioluminescence emission was maximal with 300 μg of D-luciferin.

**Conclusions:**

The results of this study suggested that the investigation of factors influencing bioluminescence measurements is a critical step toward developing a new bioluminescence imaging model. This study is the first to demonstrate that reporter genes can be transferred to intact granulosa cells with a lipid-mediated gene transfer method within intact follicles *in vitro*, and the level of transgene expression can be assessed by bioluminescence imaging in living intact antral follicles.

## Background

The ovarian antral follicle consists of an oocyte and surrounding follicular components, including theca, granulosa, and cumulus cells, and follicular fluid. It is an essential microenvironment for oocyte maturation and its developmental competence; reviewed by Moor et al. [1] and Hunter [2]. In order to investigate cellular and molecular events during the oocyte maturation and to determine factors influencing oocyte quality, several methods have been routinely used; e.g., studies of morphological characteristics, such as follicular diameter and color are usually followed by molecular and biochemical analysis of oocytes and follicular components collected from a group of follicles at defined stages of the estrous cycle. For example, the amount of protein or mRNA of estrogen receptors in lysed granulosa cells has been determined through Western blotting or qRT-PCR to assess and investigate the role of estrogen receptors in follicular development and oocyte maturation [3,4]. The estrogen contents in follicular fluid or in granulosa cell culture medium were subsequently measured. However, these methods do not provide information regarding whether functional specific genes are present or active since proteins often require activation (e.g., phosphorylation and methylation), and mRNA is often degraded during the RNA processing and transport prior to its translation into protein [5]. With recent advances in gene transfer methods, many laboratories have used reporter gene technologies to study functional gene activity and transcription factor interactions in signal transduction pathways involved in follicular atresia and steroidogenesis in cultured granulosa cells *in vitro*[6-9]. Although these studies have offered the opportunity to elucidate gene functions and cellular pathways within cultured granulosa cells, it does not take into account major interactions between the oocyte and the follicular components in the context of the whole follicle. Moreover, results from these studies usually rely on static measures using granulosa cell and follicle tissue lysates or immunohistochemistry of follicle sections. Therefore, in this study, we determine the potential to develop a new methodology to measure changes in cellular and molecular events during the oocyte maturation in the context of the living intact follicular environment.

First, we use an *in vitro* intact antral follicle culture system. It has been developed and used for large antral follicle culture in domestic animals since the 1970s. This culture system has been applied towards understanding the regulation of follicular steroidogenesis [10-13] and determining the intrafollicular factors that influence nuclear and cytoplasmic oocyte maturation [14] within intact follicles. The advantage of the intact follicle culture system over *in vitro* oocyte maturation or granulosa cell cultures is that it offers an intact microenvironment to that of the oocyte in the follicle *in vivo*, and it allows investigating interactions between the oocyte and other intrafollicular components of follicles [1,2,15]. Second, bioluminescence imaging is used in this study, to facilitate analysis of cellular and molecular events in intact living antral follicles. Bioluminescence imaging within organisms or tissues is a powerful tool that has been used in a myriad of applications for monitoring cellular signaling pathways, gene expression and regulation, protein-protein interactions and disease progression in small animals. Bioluminescence imaging is based on the detection of visible light produced by a chemical reaction, such as luciferase and its substrate, D-luciferin, in the presence of oxygen and ATP. Therefore, only live cells emit bioluminescence, and it serves also as an indicator for cell viability; reviewed by Badr and Tannous [16] and Welsh and Kay [17]. Firefly luciferase is the most commonly used bioluminescent reporter gene. A variety of molecular events in living cells, tissues, and animals can be monitored depending on the genetic regulatory sequences of interest driving the luciferase reporter gene expression [18]. Bioluminescence imaging has advantages over other systems due to its lower background as well as greater signal-to noise ratio compared to fluorescence [19]. In addition, for real-time imaging in living systems, the substrate, D-luciferin, is known to be non-toxic to mammalian cells and small animals, and luciferase has a short half-life and does not require excitation of the probe to quantify activity, which allows for dynamic measurements [20,21].Therefore, bioluminescence imaging has a great potential to determine functional gene activity and transcription factor interactions that may play essential roles in oocyte maturation and developmental competence in living intact ovarian follicles.

In this study, we explored the quantitative bioluminescence imaging of luciferase reporter-mediated gene transcription in intact living antral follicles from porcine ovaries. Bioluminescence measurements can be influenced by a variety of factors, including the time courses of luciferase-luciferin reactions, and effective plasmid DNA and D-luciferin doses and combinations [22-24]. Therefore, first, we analyzed the time course of bioluminescence emitted from transfected living intact follicles using a cationic lipid mediated gene transfer method with increasing doses of plasmid DNA (pGL4) containing a luciferase reporter gene under control of a constitutively active promoter. Second, a standard luciferase assay was used to confirm the luciferase expression in granulosa cells in the transfected intact antral follicles. Finally, the dose effects of substrate, D-luciferin, were determined for optimal quantitative bioluminescence imaging of intact porcine antral follicles *in vitro*.

## Methods

### Porcine ovary collection and antral follicle dissection

**Figure 1:**
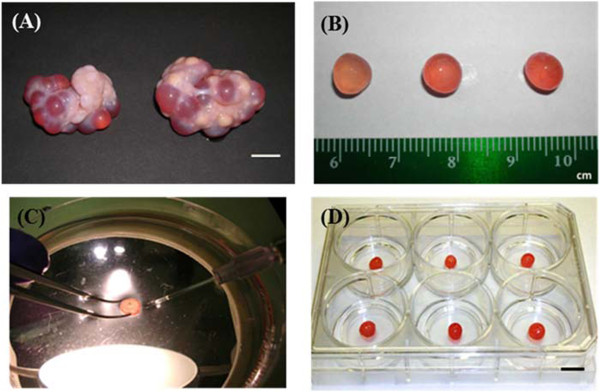
**Porcine ovaries, dissected antral follicles and DNA lipid complex microinjection of ovarian antral follicles.** A pair of porcine ovaries in follicular phase of the estrous cycle **(A)**; dissected porcine antral follicles between 6.5 to 7.5 mm in diameter **(B)**; DNA lipid complex microinjection into an antral follicle size between 6.5 to 7.5 mm in diameter on 100 × 15 mm sterile polystyrene disposable petri dish containing approximately 20 ml of CO_2_ Independent Medium using the Eppendorf FemtoJet® microinjector under a standard dissecting microscope **(C)**; and representative *in vitro* antral follicle culture condition on the membrane of Millicell-CM culture plate insert in the 6-well plate with Opti-MEM®-I reduced serum medium **(D)**. Scale bars **(A)** and **(D)** = 1.0 cm.

All of the culture medium and its supplements used in the present study were purchased from Invitrogen (Carlsbad, CA) unless otherwise indicated. Ovaries in the late follicular phase of the estrous cycle were collected from mature sows at a local slaughterhouse. The stage of estrous cycle was predicted based on the morphological description of [25]. An example of porcine ovaries collected from a mature sow is shown in Figure 1A. The ovaries were transported on ice to the laboratory within 3 hours in a sterile polypropylene container (Corning, Lowell, MA), containing 200 ml of ice cold CO_2_ Independent Medium supplemented with L-glutamin (4 mM), penicillin (100 U/ml), and streptomycin (100 μg/ml), which was also used as a dissection medium. On arrival at the laboratory, ovaries were rinsed in ice cold phosphate-buffered saline and kept in the sterile beaker with fresh dissection medium on ice. Porcine intact follicles were dissected as described by [26]. Briefly, antral follicles between diameters of 6.5 to 7.5 mm were dissected from the surrounding connective tissue of the ovary using a dissecting knife with disposable blades. These dissected follicles were trimmed of any remaining connective tissue and stromal cells using a pair of scissors and forceps (Fine Science Tools Inc., Foster City, CA) under a dissecting microscope. All the follicles were kept on ice with the dissection medium in 24-well culture plates during the entire dissection. Healthy follicles were selected on the basis of morphological criteria as described previously by [27,28]. Dissected porcine ovarian follicles between 6.5 to 7.5 mm in diameter are shown in Figure 1B.

### DNA complex formation and microinjection into the follicular antrum

The pGL4 [*luc*2/CMV/Hygro] vector (Promega, Madison, WI) carrying a luciferase reporter gene controlled by a cytomegalovirus (CMV) promoter and enhancer was used as a reporter plasmid throughout this study. pGL4 was transformed into One Shot® MAX Efficiency® DH5α™-T1R Competent Cells (Invitrogen, Carlsbad, CA). pGL4 plasmid was isolated from transfected cells and purified with the EndoFree Plasmid Maxi Kit (Qiagen, Valencia, CA) and eluted in distilled water to a final DNA concentration of 1.5-3 μg/μl. DNA complexes were prepared using the lipid based reagent FuGENE® 6 (Roche, Indianapolis, IN), according to the manufacturer’s protocol with modifications. The DNA lipid complexes were formed with a pGL4 (μg) to FuGENE® 6 reagent (μl) ratio of 2:5 (20 μg pGL4: 50 μl of FuGENE® 6). Briefly, 20 μg (1.5-3 μg/μl) of pGL4 was diluted in 50 μl of distilled water. The diluted pGL4 was transferred into a 500 μl sterile microcentrifuge tube. Fifty μl of FuGENE® 6 reagent were added to diluted pGL4, and the 100 μl of the lipid complex mixture was mixed thoroughly by brief vortexing and incubated for 20 min. The DNA lipid complex volume of 5 μl, 10 μl, and 15 μl DNA was injected into individual follicles in 1 μg, 2 μg, and 3 μg groups, respectively while the concentration of DNA lipid complexes was held constant.

For the injection of DNA complexes into the follicular antrum, a FemtoJet® (Eppendorf, Hauppauge, NY) microinjector was used in combination with Femtotips II (Eppendorf, Hauppauge, NY) injection capillaries under a standard dissecting microscope. Isolated follicles were kept for 4 hours at 38.5°C in an incubation containing 45% O_2_, 50% N_2_, and 5% CO_2_ prior to starting the microinjection procedure. The follicles were transferred individually to a 100 × 15 mm sterile polystyrene disposable petri dish (Fisher Scientific, Pittsburgh, PA) containing approximately 20 ml of CO_2_ Independent Medium (Invitrogen, Carlsbad, CA). Five μl, 10 μl, and 15 μl of DNA complex were then loaded into an injection capillary (Femtotip II) using a Microloader (Eppendorf, Hauppauge, NY) pipette tip inserted through the back of the injection capillary. Each tip of the injection capillary was broken slightly by gently pressing the tip against the edge of the fine forceps so that the DNA complex could pass through easily without clogging. Individual follicles were held by fine forceps, and the injection capillary tip was pushed into the antrum of the follicle. Then, DNA lipid complexes were injected slowly by pushing the injection button manually with an injection pressure range from 100 to 150 hPa, depending on the inner diameter of the injection capillary. The procedure of microinjection into an antral follicle is shown in Figure 1C. After injection the capillary was quickly withdrawn from the follicle to avoid introducing air into the follicle. The injection of each follicle was not conducted more than once to avoid losing follicular fluid. After the injection, the follicles were incubated for 20 hours at 38.5°C with 45% O_2_, 50% N_2_, and 5% CO_2_.

### *In vitro* intact follicle culture

Opti-MEM®-I reduced serum medium supplemented with 1% (v:v) of Insulin-Transferrin-Selenium-X Supplement (100X), penicillin (100 U/ml), and streptomycin (100 μg/ml) (Invitrogen, Carlsbad, CA) was used for the intact follicle culture. The follicles were cultured individually on a membrane of Millicell-CM culture plate insert (Cat. No. PICM0RG50; Millipore, Billerica, MA) in tissue-culture treated 6 well culture plates (Fisher Scientific, Pittsburgh, PA). The intact follicle culture condition *in vitro* is shown in Figure 1D. The insert was rested in 1.1 ml of culture medium in order to cover the surface of the follicle with a thin film of culture medium. This culture system was used to prevent the follicle surface from dryness and expose it to the air for sufficient oxygen diffusion to the inside of the follicular cells. The antral follicles were cultured at 38.5°C in a Modular Incubator Chamber (Billups-Rothenberg Inc., Del Mar, CA) containing 45% O_2_, 50% N_2_, and 5% CO_2_[10,29,30] for 20 hours.

### Bioluminescence imaging

Transient luciferase expression from a transfected porcine intact follicle was monitored 20 hours after DNA:lipid complex injection. Bioluminescence emitted from the intact follicle was detected using a Xenogen IVIS 100 Imaging system (Caliper Life Sciences, Hopkinton, MA). Ten μl of XenoLight RediJect D-luciferin (30 μg/μl in PBS; Caliper Life Sciences, Hopkinton, MA) was injected to each follicle with the microinjection method as described previously.

After injection, each intact follicle was immediately placed into a light-tight and temperature controlled chamber (39°C). Bioluminescent Images were acquired with a 30 seconds exposure. For time-course studies, images were acquired every 30 seconds for 10 minutes using a sequential image capture mode with medium binning. A constant size, circular region of interest (ROI; 1.5 cm in diameter) was drawn on the capture images over each follicle unit, and luminescence emitted from each unit was quantified using Living Image software, version 3.0 (Caliper Life Sciences, Hopkinton, MA). The signal intensity was reported as normalized total photon flux (photons/sec; p/s) within the ROI.

### Granulosa cell isolation and luciferase assay

Individual follicles were transferred into a sterile 1.5 ml microcentrifuge tube and cut in half using scissors. The follicular fluid of each follicle was isolated by centrifugation for 10 min at 1,500 × *g* at 4°C. Follicles from the each group were placed into 3 ml of HBSS (Ca^2+^/ Mg^2+^ free Hank’s Balanced Salt supplemented with 0.25 mM of HEPES) medium on a 35 mm petri dish and kept on ice. Granulosa cells were isolated by scraping the inside follicle wall using a pair of small curved forceps and centrifugated for 10 min at 1,500 × *g* at 4°C. Oocytes were removed by using 40 μm nylon cell strainers (Fisher Scientific, Pittsburgh, PA). The granulosa cells were washed three times in HBSS medium, counted in a hemocytometer, and suspended in a CO_2_ Independent Medium. For luciferase assay, 2 × 10^5^ granulosa cells per well were plated into 24-well culture dish in triplicate, and Bright–Glo (Promega, Madison, WI) was used to assay luciferase activity following the instructions given by the supplier. Luminescence emission was detected and quantified using a Xenogen IVIS 100 Imaging system (Caliper Life Sciences, Hopkinton, MA).

### Experiment 1. Time courses of bioluminescence emitted from transfected intact antral follicles

To determine whether gene transfection by a cationic lipid mediated gene transfer method was dose dependent, 1, 2, and 3 μg of plasmid DNA (pGL4) carrying a luciferase reporter gene complexed with lipid transfection reagent (Fugene® 6) was injected into intact antral follicles. A total number of 87 follicles at late follicular phase were randomly distributed in four different groups (DNA only, 1 μg, 2 μg, and 3 μg groups). The DNA only group represents a group of intact follicles (n = 22) transfected with 2 μg pGL4 (a luciferase reporter) only; 1 μg group represents a group of intact follicles (n = 22) transfected with 1 μg of pGL4: lipid complexes; 2 μg group represents a group of intact follicles (n = 21) transfected with 2 μg of pGL4: lipid complexes; and 3 μg group represents a group of intact follicles (n = 22) transfected with 3 μg of DNA:lipid complex. After 20 hours, we further analyzed the time course of bioluminescence emitted from individual transfected follicles in each group by imaging them every 30 seconds for 10 minutes. The luciferase activity (photon flux; photons/sec) of each follicle was represented as mean ± SEM from nine independent experiments.

### Experiment 2. Luciferase activity in granulosa cells

To verify the luciferase expression in granulosa cells, the cells were isolated from the transfected intact antral follicles in the DNA only, 1 μg, 2 μg, and 3 μg groups and assayed for luciferase activity using a standard luciferase assay. The experiment was repeated three times.

### Experiment 3. Effect of increasing D-luciferin (substrate) dose

To investigate luciferase activities in transfected intact follicles responding to D-luciferin substrate doses, a total number of 96 follicles were transfected with 3 μg of pGL4 (luciferase reporter gene) and randomly distributed to n = 6 different D-luciferin substrate groups (5 μg, 15 μg, 75 μg, 100 μg, 150 μg, and 300 μg groups). After 20 hours transfection, increasing doses of 5 μg, 15 μg, 75 μg, 100 μg, 150 μg, and 300 μg D-luciferin were injected (10 μl volume) into each follicle. The bioluminescence from each follicle was detected using the IVIS system after 1 min of the D-luciferin injection and expressed luciferase activity was quantified (photon flux; photons/sec). The data are represented as mean ± SEM from nine independent experiments.

### Statistical analysis

For time course studies, data were analyzed by repeated measures MANOVA with Wilks’ Lambda using the StatView program (SAS Institute Inc., San Francisco, CA). For the remaining studies, data were analyzed by one-way ANOVA followed by Fisher’s PLSD test. Data are expressed as mean ± SEM. A probability of *P <* 0.05 was considered to be statistically significant.

## Results

### Experiment 1. Time courses of bioluminescence emitted from transfected intact antral follicles

**Figure 2:**
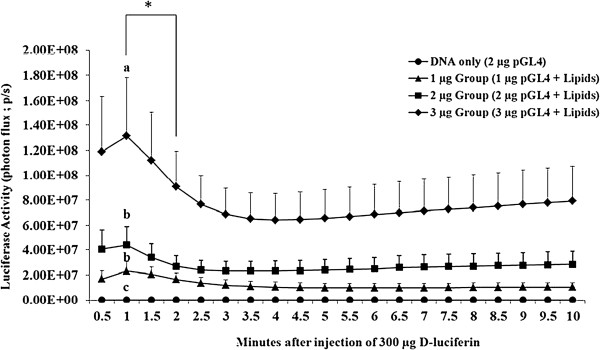
**Time courses of bioluminescence emitted from transfected intact antral follicles with increasing doses of luciferase reporter gene.** A total of n = 87 follicles were randomly distributed in four different groups (DNA only, 1 μg, 2 μg, and 3 μg groups). DNA only group represents a group of intact follicles (n = 22) transfected with 2 μg pGL4 (a luciferase reporter gene) only; 1 μg group represents a group of intact follicles (n = 22) transfected with 1 μg of pGL4: lipid complexes; 2 μg group represents a group of intact follicles (n = 21) transfected with 2 μg of pGL4: lipid complexes; and 3 μg group represents a group of intact follicles (n = 22) transfected with 3 μg of DNA:lipid complex. After 20 hours transfection, each follicle was imaged with series of 30 sec exposure time and 5 binning up to 10 minutes. Data are plotted as mean ± SEM of Luciferase Activity (Photon Flux; p/s) over time. Values with different superscripts in a group are significantly different (*P* < 0.05). *indicates significant difference (*P* < 0.05) compared to each other.

Analysis via repeated measurements showed that the luciferase activity of follicles in the 3 μg group was significantly greater than the 1 μg group (MANOVA with Wilks lambda post hoc comparison; *P* = 0.01) over time; as shown in Figure 2. There was no significant difference between the 2 μg group and 1 μg group (*P* = 0.16), or between the 3 μg group and 2 μg group (*P* = 0.12) in luciferase activity. The luciferase activity reached a peak at 1 min in the 1 μg (2.30 × 10^7^ ± 7.53 × 10^6^ p/s), 2 μg (4.38 × 10^7^ ± 1.53 × 10^7^ p/s), and 3 μg (1.31 × 10^8^ ± 4.69 × 10^7^ p/s) groups and was significantly reduced (*P* < 0.05) within 2 min after the injection of D-luciferin (Figure 2) among all groups. The mean luciferase activity of follicles in the 1 μg group, 2 μg group, and 3 μg group at 1 min was significantly reduced (*P* < 0.05) at 2 min. Luciferase activity remained constant from 2.5 to 10 min. Individual time point analysis revealed that the luciferase expression level of follicles in the 3 μg (1.31 × 10^8^ ± 4.69 × 10^7^ p/s) group was significantly greater (*P* < 0.05) than the 1 μg (2.30 × 10^7^ ± 7.53 × 10^6^ p/s) and 2 μg (4.38 × 10^7^ ± 1.53 × 10^7^ p/s) groups (one-way ANOVA with Fisher’s PLSD post hoc test) at 1 min after D-luciferin injection. In contrast, luciferase activity of intact follicles did not differ between the 2 μg and 1 μg groups (*P* = 0.62) at 1 min after luciferin injection.

### Experiment 2. Luciferase activity in granulosa cells

**Figure 3:**
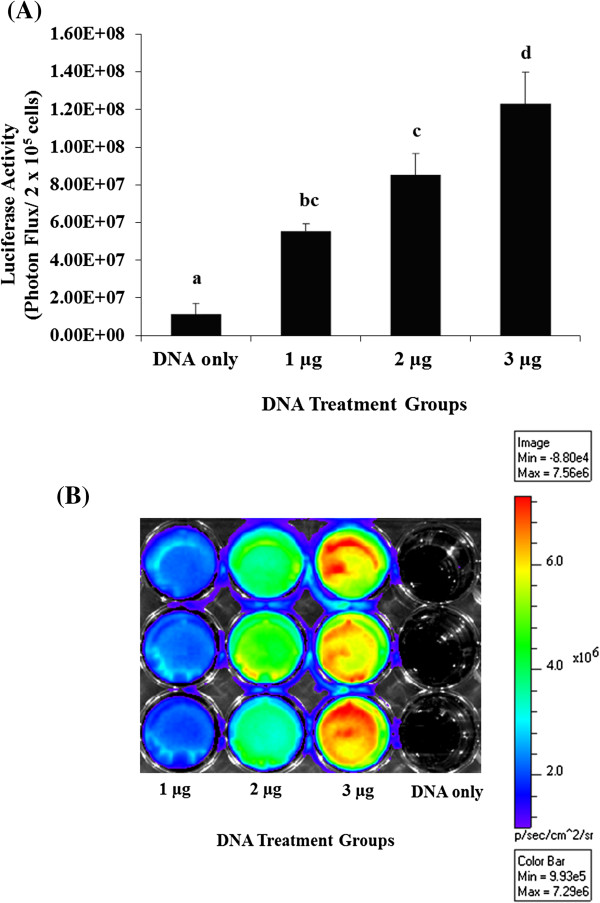
**Luciferase activities in isolated granulosa cells from intact follicles in DNA only, 1 μg, 2 μg, and 3 μg groups.** Luciferase activities in isolated granulosa cells from intact follicles in DNA only, 1 μg, 2 μg, and 3 μg groups. To demonstrate luciferase activity in follicular granulosa cells, luciferase activity of isolated granulosa cells (2 × 10^5^ cells per well) from intact follicles in the DNA only (n = 9), 1 μg (n = 8), 2 μg (n = 8), and 3 μg (n = 7) groups were quantified using standard luciferase assay (Bright-Glo) in lysed cells **(A)**. The data were expressed as mean ± SEM of Luciferase Activity (Photons Flux/2 × 10^5^ cells). Values with different superscripts in a group are significantly different (*P* < 0.05).Luminescence imaging of luciferase activity with medium binning in a 24-well plate format **(B)**. The scale bar on the right shows pseudocolor display for the photon flux of the luciferase with red and blue representing highest and lowest values, respectively. The gray scale photographic image and bioluminescence color image were superimposed.

The luciferase level of granulosa cells from the 3 μg group (1.23 × 10^8^ ± 1.69 × 10^7^ p/s) was significantly higher (*P* < 0.05) than the granulosa cells from the 2 μg group (8.52 × 10^7^ ± 1.13 × 10^7^ p/s), 1 μg group (5.53 × 10^7^ ± 3.78 × 10^6^ p/s), and DNA only group (1.12 × 10^7^ ± 5.72 × 10^6^ p/s) (Figure 3A and 3B).

### Experiment 3. Effect of increasing D-luciferin (substrate) dose on the level of luciferase in intact antral follicles

**Figure 4:**
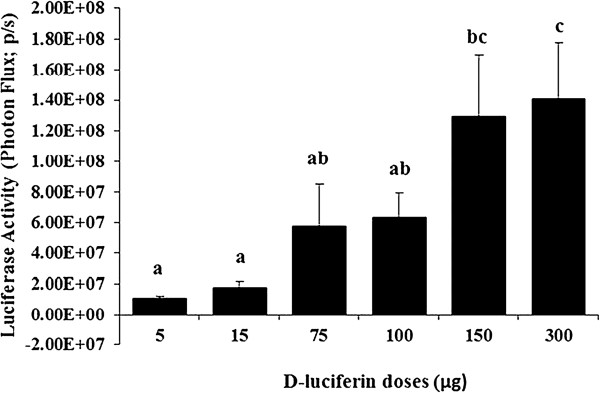
**Effect of increasing D-luciferin (substrate) doses on luciferase activity in intact antral follicles.** A total number of 96 follicles were transfected with 3 μg of pGL4 and randomly distributed to different D-luciferin substrate groups (5 μg, 15 μg, 75 μg, 100 μg, 150 μg, and 300 μg groups). After 20 hours of transfection, increasing doses of 5 μg, 15 μg, 75 μg, 100 μg, 150 μg, and 300 μg D-luciferin were injected (10 μl volume) to each follicle. The bioluminescence signal intensity was reported as mean ± SEM of luciferase activity (photon flux; photons/sec). Different letters in a group represent the luciferase activities that are significantly different (*P* < 0.05).

**Figure 5:**
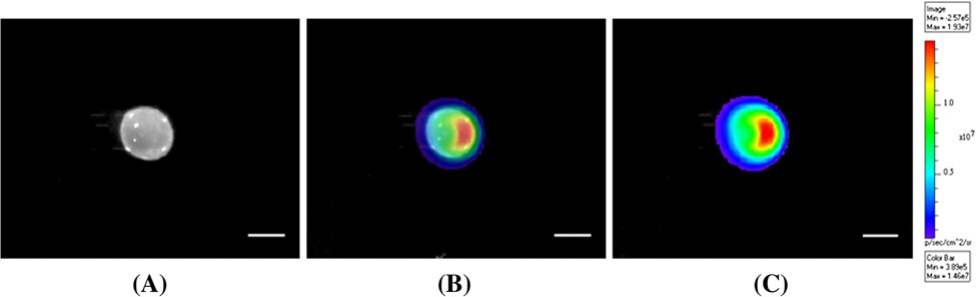
**Bioluminescence imaging of a representative follicle injected with 3 μg of pGL4 at 1 min after 300 μg of D-luciferin injection.** The gray scale photographic image of an intact ovarian follicle is shown in **(A)**. The gray scale photographic image and bioluminescence color image were superimposed with 50% of opacity, showing bioluminescence emitted from the intact follicle **(B)**. Bioluminescence signals are displayed in pseudocolors and superimposed on the gray scale photographic image. The scale bar on the right shows pseudocolor display for the photon counts of firefly luciferase with red and blue representing the highest and lowest values, respectively **(C)**. Scale bars = 0.5 cm.

We found increased luciferase activities in intact follicles with increasing doses of D-luciferin from 5 to 300 μg as shown in Figure 4. The luciferase activity was significantly greater (*P* < 0.05) in the 300 μg (1.40 × 10^8^ ± 3.77 × 10^7^ p/s) group when compared to all D-luciferin amounts, except for the 150 μg (1.29 × 10^8^ ± 4.07 × 10^7^ p/s) group. When compared between the 100 μg (6.28 × 10^7^ ± 1.63 × 10^7^ p/s) and 150 μg (1.29 × 10^8^ ± 4.07 × 10^7^ p/s) groups, no differences (*P* > 0.05) were found. The representative bioluminescence imaging of intact follicles transfected with 3 μg of luciferase reporter plasmid at 1 min after 300 μg of D-luciferin (30 μg/μl in PBS) injection is shown in Figure 5.

## Discussion

In the present study, we have demonstrated the development of a new methodology for measuring luciferase transgene expression in porcine antral follicles *in vitro* using an intact follicle culture system and bioluminescence imaging. To transfer the firefly luciferase reporter gene (pGL4) into intact follicles, we used a cationic lipid gene transfer method, which is a commonly used noninvasive, nonviral gene transfer method [31]. To our knowledge, this is the first report of bioluminescence imaging of living intact antral follicles *in vitro*. Because of the unique structure of ovarian follicles and their transparency, we were able to quantify reporter luciferase gene expression in intact follicles without disassembling the structure or lysing cells. The intrafollicular pressure of antral follicles at follicular stage is known to be low [32], thus it was possible to inject the lipid DNA complexes and D-luciferin (volume up to 15 μl) into each follicle *in vitro* without causing leakage of follicular fluid or disrupting the structure of follicles. Even after 20 hours of culture *in vitro*, and D-luciferin injection, the structure of lipid DNA complex injected follicles remained intact (See Figure 5A).

Bioluminescence imaging is based on the reaction in which firefly luciferase, produced from the introduced *luc* gene, catalyzes the oxidation of D-luciferin substrate in the presence of ATP and oxygen to produce visible light. For accurate quantification and reproducible analysis, it is necessary to determine factors that influence the intensity of bioluminescence, such as the time course of the reaction, dose responses of luciferase reporter gene after D-luciferin injection, and the dose of D-luciferin needed for optimal catalyzation reaction and light production [22-24]. First, we analyzed the time course of bioluminescence emitted from transfected intact follicles. The luciferase activity reached peak at 1 min and then was significantly reduced within 2 min. It remained constant from 2.5 min to 10 min after D-luciferin injection. The initial rise of bioluminescence from 0 min to 1 min could represent the mixing or distribution time of luciferase and D-luciferin injected in the follicles. In addition, the maximum luciferase activity at 1 min is probably the result of light produced from accumulated luciferase and D-luciferin. Because the half-life of luciferase in live mammalian cells is 2 hours [21], it can be speculated that some of the luciferase may be accumulated in the follicles during the incubation period. Following this, luciferase activity decreased within 2 min as the accumulated luciferase in the follicle was cleaved by the substrate D-luciferin. The sustained luciferase activity from 2.5 to 10 min after D-luciferin injection may represent turnover rates of the enzyme in real-time [33]. Therefore, after D-luciferase injection, bioluminescence imaging data can be analyzed at 1 minute or after 2.5 minutes for accurate quantification and reproducible data analysis. Based on this time course study, luciferase activities were acquired at 1 minute after luciferin injection in our subsequent studies with intact antral follicles.

We also determined whether gene transfection by a cationic lipid mediated gene transfer method was dose dependent, 1, 2, and 3 μg of plasmid DNA carrying a luciferase reporter gene complexed with lipid transfection reagent. We found that luciferase activity increased with increasing doses of the luciferase reporter gene. The higher luciferase activity was observed in follicles transfected with 3 μg of pGL4. A further increase in luciferase activity may be achieved by increasing the pGL4 dose above 3 μg; however, due to the limitation of the injection volume into individual intact follicles (15 μl), the 3 μg of plasmid DNA was the maximum amount injected per follicle. A large amount of transfection reagents can be toxic to mammalian cells and decrease transfection efficiencies [34]; however, we have not found any indication of toxicity in 3 μg group when compared to the lower doses 1 and 2 μg of plasmid DNA groups. Moreover, standard luciferase assays with lysed granulosa cells isolated from transfected intact follicles confirmed that luciferase expression was induced in granulosa cells in the antral follicles. The result was consistent with the results in quantitative bioluminescence imaging of living intact follicles. These findings indicated that the luciferase reporter gene can be transferred and expressed in porcine granulosa cells in an intact ovarian follicle by cationic-lipid mediated gene transfer.

In order to assess gene expression level or transcription factors using the luciferase reporter gene, it is critical to inject an excess amount of D-luciferin per follicle so that the intensity of bioluminescence is proportional to the amount of the luciferase reporter gene expressed in the follicles [24,35]. We determined the dynamics of bioluminescence in transfected follicles responding to varying D-luciferin doses and showed that the D-luciferin dose influenced the intensity of bioluminescence in intact follicles. Luciferase activity with 300 μg of substrate was significantly greater when compared to lower doses except for 150 μg. This indicated that 300 μg may be the saturating amount of D-luciferin for our experimental model. Increasing the D-luciferin dose above 300 μg may produce a further increase in light intensity; however 300 μg is the maximum amount of D-luciferin that can be injected per follicle, and use of a large amount of D-luciferin for imaging can become costly. Therefore, we used 300 μg of D-luciferin per follicle in subsequent studies.

We have demonstrated the potential to measure firefly luciferase transgene expression in granulosa cells of intact porcine antral follicles *in vitro*. We used an *in vitro* intact follicle culture system, which maintains hormonal responsiveness and physiological functions of a follicle unit resembling the *in vivo* condition. In addition, bioluminescence imaging technology provides a quantitative analysis of reporter gene expression in living intact follicles *in situ* as opposed to in cell and tissue lysates or tissue sections of follicles *ex vivo*. We have established a time course of plasmid DNA: cationic lipid dose response of luciferase gene expression, and the effect of D-luciferin amount as a first step towards a working bioluminescent imaging system for intact antral follicles. The results in this study indicated that the factors that influence the intensity of bioluminescent emissions including the time courses of luciferase-luciferin reactions, and effective plasmid DNA and D-luciferin doses and combinations are important considerations for quantitative bioluminescence imaging. These factors are critical in the development of various experimental models (e.g., *in vitro* cultured cell, tumor tissue or mouse models) and should be determined for accurate data analysis in future studies.

The present study has not addressed effects of gene delivery methods, bioluminescence imaging, and long term *in vitro* culture conditions on ovarian follicles. We are currently pursuing studies to optimize nonviral gene delivery methods (e.g., lipid-mediate and electroporation) and assess their effects and D-luciferin on granulosa cell viability, *in vitro* oocyte maturation and fertilization, and steroid content of follicular fluid in transfected intact follicles. It will be important to determine factors influencing quantitative bioluminescence and effects of gene delivery methods on intact ovarian follicles prior to applying this methodology to the functional gene analysis. Moreover, two-dimensional bioluminescence imaging used in the present study lacks depth information, and a certain area of the intact ovarian follicle in Figure 5C appears to have higher luciferase activity than others. We are speculating that it may be the site where the oocyte cumulus complex is located in the follicle, or it may just imply that a higher transgene expression was occurring within a certain area of the follicle. In the future, it will be important to investigate the structure inside of intact follicle using spatial resolution of three-dimensional optical imaging, which provides information on spatial locations.

This study is the first to demonstrate luciferase detection and quantification, indicative of transcription, within an intact antral follicle culture *in vitro*. These approaches can be used to determine specific levels of gene expression for relevant responsive gene regulatory elements by replacing the constitutive promoter in a luciferase reporter gene construct for specific transcription factors of interest; such as estrogen and progesterone response elements and p53. In addition, a specific gene expression could be inhibited with RNA interference technology to elucidate a role of the genes that are involved in oocyte maturation and developmental competence of intact antral follicles; an area of possible future research even though specific promoter driven reporter genes usually leads to a weaker gene expression compared to a constitutive promoter used in this study. Moreover, this methodology may be used as a model for the delivery of luciferase reporters carrying therapeutic genes that might encode growth hormones, steroid receptors, or anti-apoptotic factors into granulosa cells in antral follicles to support oocyte maturation and quality. Lastly, this study may assist in determining the feasibility of gene transfer within the intact follicles or the whole ovary in domestic animals *in vivo* via ovarian transvaginal ultrasonography guided fine needle injection method described in [36]. The light attenuation in tissue of large animals can be problematic. However, bioluminescence can be visualize and monitored by an endoscopic or laparoscopic bioluminescence imaging tool. Moreover, transgene expression in ovaries and follicles *in vivo* with fluorescent reporter genes can be monitored using fibered confocal fluorescence microscopy [37]. Future studies need to address the feasibility of such imaging modalities for application in domestic animals.

## Conclusions

We have demonstrated the development of a new methodology for measuring luciferase transgene expression in porcine intact follicles *in vitro* using an intact follicle culture system and bioluminescence imaging. The time course of plasmid DNA: cationic lipid dose response of luciferase gene expression, and the effect of D-luciferin amount were established as a first step towards a working bioluminescent imaging system for intact antral follicles. A higher level of luciferase expression was observed in follicles transfected with 3 μg of plasmid reporter DNA, with an optimal time for quantification at 1minute after 300 μg of D-luciferin injection per follicle. This methodology can be used to determine specific levels of functional gene expression during the oocyte maturation process within the follicle and possibly applied to gene therapy approaches to improve oocyte maturation and quality.

## Competing interests

The authors declare that they have no competing interests.

## Authors’ contributions

This study represents a part of SJ’s doctoral dissertation submitted to and accepted by the Mississippi State University. STW and SJ contributed to conception and design. SJ contributed to all experimental activities, data and statistical analysis, and interpretation of data. STW was responsible for overall supervision and supervised the project. SJ drafted the manuscript, which was revised by STW. Both authors read and approved the final manuscript.

## Authors’ information

SJ is a postdoctoral associate in the Department of Animal and Dairy Sciences, Mississippi State University. STW is a professor of Department of Biochemistry, Molecular Biology, Entomology and Plant Pathology and Department of Animal and Dairy Sciences and an associate dean of College of Agriculture and Life Sciences, Mississippi State University.

## References

[B1] Moor RM Lee C Dai YF Fulka J Jr Antral follicles confer developmental competence on oocytes Zygote 1996 4 289 293 9153767 10.1017/s0967199400003269

[B2] Hunter MG Follicular factors regulating oocyte maturation and quality Hum Fertil (Camb) 1998 1 69 74 10.1080/1464727982000198151 11844313

[B3] Bao B Kumar N Karp RM Garverick HA Sundaram K Estrogen receptor-β expression in relation to the expression of luteinizing hormone receptor and cytochrome P450 enzymes in rat ovarian follicles Biol Reprod 2000 63 1747 1755 10.1095/biolreprod63.6.1747 11090445

[B4] Jansen HT West C Lehman MN Padmanabhan V Ovarian estrogen receptor-β (ERβ) regulation: I. Changes in ERβ messenger RNA expression prior to ovulation in the ewe Biol Reprod 2001 65 866 872 10.1095/biolreprod65.3.866 11514352

[B5] Shapiro JA Revisiting the central dogma in the 21st century Ann N Y Acad Sci 2009 1178 6 28 10.1111/j.1749-6632.2009.04990.x 19845625

[B6] Benco A Sirotkin AV Vasicek D Pavlova S Zemanova J Kotwica J Darlak K Valenzuela F Involvement of the transcription factor STAT1 in the regulation of porcine ovarian granulosa cell functions treated and not treated with ghrelin Reproduction 2009 138 553 560 10.1530/REP-08-0313 19528263

[B7] Geng LY Fang M Yi JM Jiang F Moeen-ud-Din M Yang LG Effect of overexpression of inhibin alpha (1-32) fragment on bovine granulosa cell proliferation, apoptosis, steroidogenesis, and development of co-cultured oocytes Theriogenology 2008 70 35 43 10.1016/j.theriogenology.2008.02.013 18456314

[B8] Sirotkin AV Benco A Tandlmajerova A Vasicek D Involvement of transcription factor p53 and leptin in control of porcine ovarian granulosa cell functions Cell Prolif 2012 45 9 14 10.1111/j.1365-2184.2011.00793.x 22151798 PMC6496811

[B9] Wang W Chen X Li X Wang L Zhang H He Y Wang J Zhao Y Zhang B Xu Y Interference RNA-based silencing of endogenous SMAD4 in porcine granulosa cells resulted in decreased FSH-mediated granulosa cells proliferation and steroidogenesis Reproduction 2011 141 643 651 10.1530/REP-10-0098 21292728

[B10] Moor RM Sites of steroid production in ovine graafian follicles in culture J Endocrinol 1977 73 143 150 10.1677/joe.0.0730143 870585

[B11] Baker TG Hunter RH Neal P Studies on the maintenance of porcine graafian follicles in organ culture Experientia 1975 31 133 135 10.1007/BF01924721 1167516

[B12] Shemesh M Ailenberg M The effect of androstenedione on progesterone accumulation in cultures of bovine ovarian follicles Biol Reprod 1977 17 499 505 10.1095/biolreprod17.4.499 922085

[B13] Tsafriri A Lindner HR Zor U Lamprecht SA *In vitro* induction of meiotic division in follicle-enclosed rat oocytes by LH, cyclic AMP and prostaglandin E2 J Reprod Fertil 1972 31 39 50 10.1530/jrf.0.0310039 4342745

[B14] Fouladi-Nashta AA Campbell KH Dissociation of oocyte nuclear and cytoplasmic maturation by the addition of insulin in cultured bovine antral follicles Reproduction 2006 131 449 460 10.1530/rep.1.00581 16514188

[B15] Moor RM Dai Y Lee C Fulka J Jr Oocyte maturation and embryonic failure Hum Reprod Update 1998 4 223 236 10.1093/humupd/4.3.223 9741707

[B16] Badr CE Tannous BA Bioluminescence imaging: progress and applications Trends Biotechnol 2011 29 624 633 10.1016/j.tibtech.2011.06.010 21788092 PMC4314955

[B17] Welsh DK Kay SA Bioluminescence imaging in living organisms Curr Opin Biotechnol 2005 16 73 78 10.1016/j.copbio.2004.12.006 15722018

[B18] Hodge DR Clausen PA Transcriptional activation analysis using bioluminescent reporter assays Mol Biotechnol 2000 16 67 76 10.1385/MB:16:1:67 11098469

[B19] Fan F Wood KV Bioluminescent assays for high-throughput screening Assay Drug Dev Technol 2007 5 127 136 10.1089/adt.2006.053 17355205

[B20] Contag PR Olomu IN Stevenson DK Contag CH Bioluminescent indicators in living mammals Nat Med 1998 4 245 247 10.1038/nm0298-245 9461201

[B21] Ignowski JM Schaffer DV Kinetic analysis and modeling of firefly luciferase as a quantitative reporter gene in live mammalian cells Biotechnol Bioeng 2004 86 827 834 10.1002/bit.20059 15162459

[B22] Burgos JS Rosol M Moats RA Khankaldyyan V Kohn DB Nelson MD Jr Laug WE Time course of bioluminescent signal in orthotopic and heterotopic brain tumors in nude mice Biotechniques 2003 34 1184 1188 12813886 10.2144/03346st01

[B23] Cui K Xu X Zhao H Wong ST A quantitative study of factors affecting *in vivo* bioluminescence imaging Luminescence 2008 23 292 295 10.1002/bio.1032 18452141

[B24] Virostko J Jansen ED Validation of bioluminescent imaging techniques Methods Mol Biol 2009 574 15 23 10.1007/978-1-60327-321-3_2 19685296

[B25] Hunter FH Baker TG Development and fate of procine Graafian follicles identified at different stages of the oestrous cycle J Reprod Fertil 1975 43 193 196 10.1530/jrf.0.0430193 1168709

[B26] Moor RM Hay MF McIntosh JE Caldwell BV Effect of gonadotrophins on the production of steroids by sheep ovarian follicles cultured *in vitro* J Endocrinol 1973 58 599 611 10.1677/joe.0.0580599 4795508

[B27] Kruip TA Dieleman SJ Macroscopic classification of bovine follicles and its validation by micromorphological and steroid biochemical procedures Reprod Nutr Dev 1982 22 465 473 10.1051/rnd:19820403 6891491

[B28] Guthrie HD Grimes RW Cooper BS Hammond JM Follicular atresia in pigs: measurement and physiology J Anim Sci 1995 73 2834 2844 8582874 10.2527/1995.7392834x

[B29] Bryant-Greenwood GD Jeffrey R Ralph MM Seamark RF Relaxin production by the porcine ovarian graafian follicle *in vitro* Biol Reprod 1980 23 792 800 10.1095/biolreprod23.4.792 7448280

[B30] Fouladi Nashta AA Waddington D Campbell KH Maintenance of bovine oocytes in meiotic arrest and subsequent development *In vitro*: a comparative evaluation of antral follicle culture with other methods Biol Reprod 1998 59 255 262 10.1095/biolreprod59.2.255 9687293

[B31] Gao X Kim KS Liu D Nonviral gene delivery: what we know and what is next AAPS J 2007 9 E92 E104 10.1208/aapsj0901009 17408239 PMC2751307

[B32] Ahmed Ebbiary NA Lenton EA Welsby R Mowforth A Cooke ID Folliculocentesis: a novel research technique to investigate the intrafollicular endocrine microenvironment Hum Reprod 1995 10 2325 2333 10.1093/oxfordjournals.humrep.a136294 8530661

[B33] Wood KV The chemistry of bioluminescent reporter assays Promega Notes 1998 65 14

[B34] Luo D Saltzman WM Enhancement of transfection by physical concentration of DNA at the cell surface Nat Biotechnol 2000 18 893 895 10.1038/78523 10932162

[B35] Berger F Paulmurugan R Bhaumik S Gambhir SS Uptake kinetics and biodistribution of 14C-D-luciferin–a radiolabeled substrate for the firefly luciferase catalyzed bioluminescence reaction: impact on bioluminescence based reporter gene imaging Eur J Nucl Med Mol Imaging 2008 35 2275 2285 10.1007/s00259-008-0870-6 18661130 PMC4157642

[B36] Ginther OJ Bergfelt DR Beg MA Meira C Kot K *In vivo* effects of an intrafollicular injection of insulin-like growth factor 1 on the mechanism of follicle deviation in heifers and mares Biol Reprod 2004 70 99 105 12954722 10.1095/biolreprod.103.021949

[B37] Velazquez MA Kues WA Komorowska MA, Olsztynska-Janus S *In vivo* gene transfer in the female bovine: potential applications for biomedical research in reproductive sciences Biomedical engineering, trends, research and technologies 2011 In Tech 217 244 [http://www.intechopen.com/books/biomedical-engineering-trends-research-and-technologies/in-vivo-gene-transfer-in-the-female-bovine-potential-applications-for-biomedical-research-in-reprodu]

